# HCV and HIV co-infection among people who inject drugs in Vietnam

**Published:** 2020-12

**Authors:** Vu Toan THINH, Li LI, Dréan MATTHIEU, Van Dinh HOA, Nguyen Huu ANH, Le Minh GIANG

**Affiliations:** 1 M.Sc, Center for Research and Training on Substance Use-HIV, Hanoi Medical University, Hanoi 100000, Vietnam.; 2 Professor, Semel Institute for Neuroscience and Human Behavior - Center for Community Health, University of California, Los Angeles, CA 90024, USA.; 3 Médecins du Monde in Vietnam, Hanoi 100000, Vietnam.; 4 M.D., M.P.H, Center for Research and Training on Substance Use-HIV, Hanoi Medical University, Hanoi 100000, Vietnam.; 5 M.D., Associate Professor, Center for Research and Training on Substance Use-HIV, Hanoi Medical University, Hanoi 100000, Vietnam.

**Keywords:** HIV/HCV co-infection, HCV mono-infection, People Who Inject Drugs, Vietnam

## Abstract

**Introduction:**

HIV/HCV co-infection in people who inject drugs (PWID) continues to be a major challenge for health care systems and the PWID themselves. PWID have driven the HIV epidemic in Vietnam but information on HIV/HCV co-infection is limited.

**Methods:**

A cross-sectional study was conducted with 509 PWID recruited in Hanoi from February 2016 to April 2017. Four mutually exclusive groups were defined based on the presence of detectable HCV RNA and positive HIV confirmation. Multiple logistic regression analyses were performed to explore life-time risk behaviors of HCV mono-infection and HIV/HCV co-infection.

**Results:**

The overall prevalence of HIV and HCV infection was 51.08% and 61.69%, respectively. The prevalence of HCV mono-infection and HIV/HCV co-infection was 22.59% and 39.1%, respectively. We found that engaging in methadone maintenance treatment (MMT) was positively associated with HCV mono-infection (aOR = 2.38, 95% Confidential Interval [CI] 1.07 to 5.28) and with at least either HIV or HCV infection (aOR = 2.22, 95% CI 1.08 to 4.56). Ever being incarcerated was significantly associated with HCV mono-infection (aOR = 2.56, 95% CI 1.33 to 4.90) and HIV/HCV co-infection (aOR = 1.90, 95% CI 1.04 to 3.46). Those who had ever shared with and reused syringes/needles were more likely to have HIV/HCV co-infection (aORs = 5.17 and 2.86, *P* < 0001, respectively) and have either HIV or HCV infection (aORs = 3.42 and 2.37, *P* < 0001, respectively).

**Conclusion:**

Correlates for HCV mono-infection and HIV/HCV co-infection highlight the need to address risk behaviors, expand MMT programs, and establish HCV sentinel surveillance. The high prevalence of HCV and/or HIV co-infection shows a need for access to HCV treatment.

## Riassunto

**Introduzione:**

La coinfezione da HIV/HCV nei tossicodipendenti continua ad essere una sfida importante per i servizi sanitari e gli stessi tossicodipendenti. Questi ultimi hanno diffuso l’infezione da HIV nel Vietnam, ma l’informazione sulla co-infezione da HIV/HCV è limitata.

**Metodi:**

Uno studio trasversale è stato condotto con 509 tossicodipendenti reclutati ad Hanoi dal febbraio 2016 ad aprile 2017. Quattro gruppi mutualmente esclusivi sono stati definiti sulla base della presenza di riscontro di HCV RNA e di conferma di sieropositività per HIV. È stata effettuata l’analisi di regressione logistica per esplorare i comportamenti a rischi nel corso della vita per monoinfezione da HCV e coinfezione da HIV-HCV.

**Risultati:**

La prevalenza complessiva dell’infezione da HIV ed HCV è stata del 51.08% e del 61.69%, rispettivamente, mentre quella della monoinfezione da HCV e della coinfezione da HIV/HCV del 22.59% e del 39.1%. Abbiamo scoperto che essere sottoposti a trattamento di mantenimento con metadone (MMT) è risultato positivamente associato con la monoinfezione da HCV (aOR = 2.38, 95% IC 1.07–5.28) e con almeno o l’infezione da HIV o con quella da HCV (aOR = 2.22, 95% IC 1.08–4.56). Una storia di prigionia è stata associata in modo significativo all’infezione da HCV (aOR = 2.56, 95% IC 1.33–4.90) ed alla co-infezione da HIV/HCV (aOR = 1.90, 95% IC 1.04–3.46). Quelli che avevano sempre condiviso o riutilizzato aghi/siringhe avevano una maggiore probabilità di avere una coinfezione da HIV/HCV (aORs = 5.17 e 2.86, *P* < 0001, rispettivamente) ed una infezione da HIV o HCV (aORs = 3.42 e 2.37, *P* < 0001, rispettivamente).

**Conclusione:**

I correlati di monoinfezione da HCV e di co-infezione da HIV/HCV evidenziano la necessità di affrontare i comportamenti a rischio, espandere i programmi terapeutici di mantenimento con il metadone ed instaurare una sorveglianza sentinella per l’HCV. L’alta prevalenza di HCV e/o di co-infezione da HIV mostra la necessità di accedere al trattamento per l’HCV.

## INTRODUCTION

HIV/HCV co-infection is a burgeoning public health issue with the burden among people who inject drugs (PWID) significantly higher than other at-risk groups and further complicated by a lack of prevention interventions for PWID [1]. More than half of the global estimates of HIV/HCV co-infection are PWID [2, 3]. In the South and Southeast Asia, this prevalence varied from 65.8% to 96.1%, with the highest proportions in Nepal and Vietnam [2]. Vietnam had a large proportion of PWID with approximately 200,000 persons [4], and a high prevalence of HCV reported at approximately 87% among this high-risk population [5]. It is important to note that HIV-infected people have six times the odds of HCV infection higher than in HIV-negative population [2]. A study recruited people living with HIV/AIDS in 30 provinces and cities in Vietnam showed that the prevalence of HBV, HCV, and HIV coinfection accounted for 50.3% [6]. However, there is no recent data on prevalence of HIV/HCV co-infection and HCV mono-infection among Vietnamese PWID since the last survey was reported by the government’s 2013 Integrated Biological and Behavioral Assessment (IBBS) [7].

It is well-known that HIV/HCV co-infection in PWID presents a variety of challenges in clinical care, treatment, and the prevention of morbidity and mortality [6–10]. Health investment on co-infections among PWID in developing countries is not comparable with the burden that HIV/HCV co-infection has caused [1]. Therefore, identification of the prevalence of HIV/HCV co-infection among PWID is important to reduce the burden of disease and provide an evidence base for effective intervention programs.

Previous studies, conducted in developed countries, have identified important risk factors associated with current or previous HIV/HCV co-infection and HCV mono-infection, including socio-demographics and risk behaviors in comparison to non-infection groups [1, 6, 10–12]. These studies identified significant factors associated with HCV mono-infection, including a history of drug injection [10], a history of sharing syringes within six months ahead of signing up for methadone treatment program (MMT), prior incarceration [11], living alone or having a spouse/partner without children, and being homeless [13]. Factors relating to HIV/HCV co-infection included ever sharing diazepam or novocaine [1], residence in urban areas, averaging greater than three injections per day in the past 30 days [10], being a man who has sex with other men, not completing high school [12], and previous incarceration [11]. This is not the case in developing countries that focused on estimating the prevalence of past or current HCV or HIV mono-infection in rural settings rather than focusing on identifying factors associated with current HCV infection and HIV/HCV co-infection [1, 14–16]. Given this data, discovering the factors that impact the infection status in lowand middle-income countries with limited resources, especially in countries that are receiving less international aid for HIV control and treatment such as Vietnam, is essential. Faced with a high prevalence of HIV/HCV co-infection and the severe consequences to the population’s health, interferon-free direct-acting antivirals (DAA) are proven to revolutionize HCV therapeutics. However, reinfection amongst PWID continues to be the most frequent compared to other at-risk populations [17, 18]. More importantly, the costs for a 12-week course of HCV treatment are approximately $ 60,000 – $ 80,000 making the treatment unavailable to the most PWID [19]. Given these situations, the Hanoi medical University implemented the first pilot study assessing the stages of HCV infection as well as providing effective intervention models for counseling, early detection, and prompt treatment referral for PWID in Hanoi, Vietnam. Therefore, this paper aims to understand the association between lifetime risk behaviors and current HCV mono-infection and HIV/HCV co-infection among PWID in Hanoi, Vietnam. The information on identifying the sources of contributing factors helps intervention programs identify at high-risk individuals, which is deemed essential towards reducing the burden of the disease in developing countries.

## METHODS

Study design, population, and data collection This cross-sectional study was conducted between February 2016 and April 2017 in Hanoi, Vietnam. Peer-educators from five community-based organizations were responsible for outreaching to PWID at hotspots in 11 districts, screening for eligibility, and providing study information for informed consent to be enrolled in the study. Eligible participants met the following criteria: (1) being at least 18 years old, (2) self-reported injection at least once in the lifetime, (3) currently living in Hanoi, and (4) voluntarily agreeing to participate in the study. These eligible PWID were referred to the Sexual Health Promotion (SHP) clinic, Hanoi Medical University, for collecting serological and behavioral data. Out of 844 PWID who came to the SHP clinics, 328 PWID (39.7%) were excluded from this analysis (7 PWID were ineligible, and 328 PWID did not have sufficient data about their behaviors). A total of 509 PWID with complete behavioral and serological data were included in the final analysis.

### Study measures and instruments

Socio-demographic information included participant’s age, gender (male, female), marital status (single, married, divorced, and widowed), living area (urban districts, suburban districts), living arrangement (family members and others), education (graduated and not graduated from high school), employment status (employed, unemployed, students, and others), MMT treatment (yes, no) and ever being incarcerated (yes, no). HCV knowledge was assessed based on 9 true or false questions (e.g., ‘Someone who has been treated and cured for hepatitis C cannot be infected by the virus again’; ‘HCV progression is accelerated by alcohol’; etc.) and the overall score was then dichotomized into *good* (> 5 out of 9) and *not good* (≤ 5), using the average score as a cut-off.

Lifetime risk behaviors were the primary exposures of interest in this study. We assessed the frequency of 15 risk behaviors in their life using a locally developed questionnaire with a focus on ever sharing syringes/needles, ever reusing syringes/needles, ever sharing a pipe for inhaling drugs, ever having intercourse with a PWID not used condoms, having sex for money and/or drugs without using condoms, and unsafe tattooing. Items were assessed on a dichotomous scale: ‘0’ (never) or ‘10’ (yes). If participants answered ‘yes’, corresponding questions were assessed on a five-point Likert scale in the previous six months: never, 1–2 times, monthly, weekly, and almost every day. However, as the corresponding proportions of risk behaviors in the last six months remained low in this dataset, we decided to use lifetime risk behaviors as the independent variables.

HIV and HCV test results were the primary outcome of interest. HIV infection was confirmed by the Elisa test, and HCV RNA was quantified using HCV viral load testing, among PWID with HIV and/or anti-HCV positive, at the Vietnam National Hospital of Tropical Diseases.

### Statistical analysis

The data was cleaned by checking missing patterns, outliers, and invalid data before being entered in Epi Data 3.1, then cleaned and converted into a CSV file to be analyzed by SAS 13.0 (SAS Institute, Cary, NC, USA). Frequencies and percentages were presented for categorical variables. We calculated the mean and standard deviation (SD) of the distribution for normally distributed continuous variables. Simple logistic analysis was used to assess the bivariate association of each selected factor with HCV mono-infection, HIV/HCV co-infection, and at least one of the two infections. Predetermined confounders, including age group, gender, marital status, living arrangement, and HCV knowledge score, were locked into the model regardless of the significance level. The final multiple logistic models were evaluated based on (1) model calibration with Hosmer-Lemeshow goodness-of-fit (*P* > 0.05), (2) variance inflation factors (VIF) to check multi-collinearity, and (3) predictive accuracy with areas under the curve (AUC >.7).

### Ethical consideration

The de-identified data analysis was approved by the Hanoi Medical University (the host institution) and received an exemption from the University of California at Los Angeles Institutional Review Board.

## RESULTS

### Participants’ socio-demographic characteristics and life-time risk behaviors

Of the 509 participants recruited, 68% were older than 35 years old. Most of the participants were male (at 78.4%), lived with family members (81.9%), and lived in urban areas (81.3%). Approximately 14% lived with HCV individuals. Precisely 61.3% of participants did not graduate from high school, more than half of participants were currently married, and 82.7% were currently employed. Around 62% reported ever being incarcerated in their lifetime (Table 1).

Approximately 59% of participants reported ever sharing a needle or a syringe, and more than a third of participants ever reused others’ syringes and needles. Over half of the participants reported ever sharing pipes for inhaling drugs. Over one-third of PWID surveyed had sex with other PWID without condoms, and about 23% of participants ever traded intercourse for money and/or drugs without using condoms. More than 40% reported having unsafe tattooing, and about 44% ever shared personal things such as razors/nail scissors. There were 96 PWID (accounting for 18.9%) on methadone treatment at the study’s enrollment, and over half of participants had HCV knowledge scores greater than five out of a maximum of nine.

### Proportions of current HIV and HCV infection

Of all participants with serological data in HCV and HIV indicators, the overall prevalence of HIV and HCV was 51.08% and 61.69%, respectively. The prevalence of HCV mono-infection and HIV mono-infection was 22.59% and 11.98%, respectively. The prevalence of HIV/HCV co-infection was 39.1%. Approximately 26% of participants were uninfected.

### Correlates of HCV mono-infection

Bivariate analysis of HCV mono-infection showed that factors significantly and positively associated with HCV mono-infection, included being older than 35 years old, male, living with family members, and being incarcerated at least once in their lifetime, currently on methadone treatment and ever had unsafe tattooing. Risk behaviors of interest such as ever sharing syringes/needles, reusing syringes/needles, sharing pipes with others, and having sex for money and/or drugs without condoms, were not significantly associated with HCV mono-infection. Those who ever had sex with other PWID and did not use condoms and ever shared personal things like razor/nail scissors were less likely to have HCV mono-infection (Table 2).

As shown in Table 3, after controlling for potential confounders, being currently on MMT (aOR = 2.38, 95% Confidential Interval [CI] 1.07 to 5.28) and ever being incarcerated (aOR = 2.56, 95% CI 1.33 to 4.90) were both significantly associated with higher odds of HCV mono-infection. Reporting ever sharing syringes/needles (aOR = 1.12, 95% CI 0.55 to 2.27), reusing syringes/needles (aOR = 0.62, 95% CI 0.27 to 1.41), sharing pipes for inhaling drugs with others (aOR = 1.75, 95% CI 0.94 to 3.28), and ever had unsafe tattooing (aOR = 1.80, 95% CI 0.95 to 3.40) were not significantly associated with HCV mono-infection.

### Correlates of HIV/HCV co-infection

In bivariate analysis, being over 35 years old, male, living with family, being married, ever incarcerated, having a higher HCV knowledge score, and currently engaging in MMT clinics were significantly associated with higher odds of HIV/HCV co-infection. Lifetime risk behaviors such as ever shared syringes/needles, reused syringes/needles, shared pipes with others, had sex for money and drugs without using condoms, and had unsafe tattooing were associated with a higher likelihood to have HIV/HCV co-infection. Those who lived in urban districts or ever shared personal things had a lower likelihood of HIV/HCV co-infection. An interesting finding is that only ever having sex with PWID without using condoms was not significantly associated with the co-infection status.

In multivariate analysis, after adjusting for several variables, ever incarcerated (aOR = 1.90, 95% CI 1.04 to 3.06), ever shared syringes/needles (aOR = 5.17, 95% CI 2.69 to 9.93), and ever shared pipe with others (aOR = 2.86, 95% CI 1.55 to 5.29) were all significantly associated with higher odds of HIV/HCV co-infection. No statistical difference was found among those who reported ever reusing syringes/needles, having unsafe tattooing, and being currently on MMT treatment.

### Correlates of at least one infection

Table 2 shows that factors associated with higher odds of having at least one infection in the simple logistic regression included those aged 35 years and older, males, lived with their family, married, have higher HCV knowledge score, and currently engage in MMT treatment. Additionally, those who reported ever sharing syringes/needles, reusing syringes/needles, having sex with PWID without using condoms, and unsafe tattooing were more likely to have at least one infection. On the other hand, those trading sex for money and drugs without condoms and ever sharing personal things with others were less likely to have infections.

After adjusting for other variables (Table 3), being currently on MMT treatment (aOR = 2.22, 95% CI 1.08 to 4.56), and ever sharing syringes/needles (aOR = 3.42, 95% CI 2.01 to 5.82), ever sharing pipes for inhaling drugs with others (aOR = 2.37, 95% CI 1.44 to 3.87), and ever having unsafe tattooing (aOR = 1.81, 95% CI 1.09 to 3.03) were positively and significantly associated with the odds of having at least one of two infections.

## DISCUSSION

Of note, significant correlates of HIV/HCV co-infection were ever sharing syringes/needles and ever sharing pipes for inhaling drugs. The findings are consistent with the previous studies showing that sharing syringes and needles were associated with an increased risk of co-infection [20, 21]. Another interesting point is that greater than 80% of PWID in our study smoked opioids with pipes, which is not typically associated with blood exposure. However, many PWID suffer chronic burns and sores in their mouths, facilitating oral HCV transmission. Therefore, besides blood exposure prevention, we need to pay more attention to the means of using drugs that could increase the risk of HCV infection. In this study, ever sharing syringes/needles and ever sharing pipes for inhaling drugs are not significantly associated with HCV mono-infection. This may be because co-infected PWID are exposed to unsafe injecting behaviors to a greater extent than mono-infected ones [1]. Also, a history of reusing a needle did not significantly increase the likelihood of both HCV mono-infection and HIV/HCV co-infection. The result differs from another Vietnamese study that presents the odds of HIV/HCV co-infection among those who reused a syringe and a needle are more than three times higher than those without reusing them [1]. A possible explanation is that our study included both active and non-active PWID. Since we collected data on lifetime (including past and present) risk behaviors, the data could be impacted by recall bias. Therefore, the practice of reusing syringes/needles in the past might not reflect in line with the current status of infection.

In our study, we did not take the pattern of substance use and history of injection into consideration to different factors associated with HCV mono-infection and HIV/HCV co-infection. Previous studies highlighted that drug injecting history is significantly and positively associated with HCV infection status with and without HIV infection [1, 10, 13]. Co-infected PWID exhibited higher odds of injecting more than four times per day [22] and injecting for more than two years [1, 16]. Therefore, it is important to include the history of injecting when evaluating factors associated with infection status in the long run. Additionally, ever having unsafe tattooing is positively associated with the odds of having at least one infection compared to non-infection. Previous studies also reported that non-professional tattooing is a risk factor for HCV infection exposure, even among the population without traditional risk behaviors [23, 24].

After adjusting for potential confounders, we found that those who reported ever being incarcerated were more likely to have HCV mono-infection and HIV/HCV co-infection. This result is supported by ample evidence that prison environments contain hidden factors that enhance the risk of HCV infection [10, 11, 15, 25, 26]. The Vietnam Administration of HIV/AIDS Prevention and Control has issued a guideline on harm reduction interventions to prevent HIV transmission for PWID, which focuses on information, education, and communication (IEC) activities. This program provides condoms as well as clean and sterile syringes/needles, but it has yet to reach incarcerated populations [27]. Moreover, low coverage of services and availability of harm reduction materials, together with illegal injecting behaviors, may have promoted behaviors with a higher risk in this population [10]. Therefore, apart from the expansion of harm reduction services, correctional settings should be targeted for HCV screening programs and linkage to care since PWID need to be provided with HCV treatment initiation and continuation while they are in these settings [20].

Our study showed that those who are currently on MMT treatment had higher odds of HCV mono-infection and at least one of two infections. However, this factor is not significantly associated with HIV/HCV co-infection. In our research, 31.3% of HCV mono-infection reported being currently on MMT, and this increased the likelihood of HCV mono-infection by odd of 2.38 but not HIV/HCV co-infection. A study conducted in Cambodia reported that PWID receiving MMT in the last 12 months were 2.67 times more likely to have HCV mono-infection than those who did not [28]. Therefore, being in an MMT program may provide an opportunity for early identification of HCV and/or HIV infection. Previous studies also revealed that the MMT program scale-up had a substantial impact on reducing HIV and HCV incidence because it prevented exposure, for both infected and susceptible PWID, to risk behaviors [29]. In addition to MMT expansion, it is pivotal to scale up intervention programs such as HIV and/or HCV testing and harm reduction programs in these facilities. In this study, we observed a high prevalence of HCV mono-infection and HIV/HCV co-infection. However, these estimates are much lower than the global prevalence of 52.3% and 82.4%, respectively [2, 30]. In contrast, our study found that the co-infection prevalence is slightly higher than the corresponding proportion in Thai Nguyen (34.8%) and ten provinces with high HIV prevalence based on the IBBS report (at 26.3%) [1, 15]. The difference with the IBBS could be explained by the fact that this survey only recruited male PWID as well as used the median prevalence including a higher prevalence of co-infected PWID in Quang Ninh and Hai Phong (>50%) while no cases were found in Da Nang [15]. Our finding not only shows a high prevalence of HCV and HIV/HCV co-infection, but it also underscores important information on the burden of disease among PWID in urban settings. This finding is supported by ample evidence showing that PWID are at higher risk of HCV infection and the HIV/HCV co-infection also increases the progression of HIV to acquired immunodeficiency syndrome (AIDS), accelerates the course of HCV, and the risk of developing hepatic cirrhosis, hepatocellular carcinoma, and liver failure. Additionally, among those living with HIV, HCV associated end-stage liver disease is a major cause of death [10, 22]. In the context of implementing direct-acting antivirals (DAA) in Vietnam, paralleling with ART treatment, it is suggested that HCV treatment should be provided to PWID to reduce the burden of liver disease, liver cancer, and improve quality of life. The findings also highlight the importance of establishing HCV sentinel surveillance among this target population to build accurate data on HCV infection with/without HIV. This, in turn, provides evidence to support efforts to make changes in public health policy and guide programs to reduce HCV infection transmission.

There are several limitations to this study. Because of the convenience sampling, there is a likelihood that PWID knew their status and wanted to enroll in this first intervention study providing a full package from counseling, free-of-charge HCV testing, and treatment in Hanoi. Therefore, there is a possibility that this study may overestimate the true prevalence of HCV mono-infection and HIV/HCV co-infection as well as introduce selection bias. Although an effort to engage PWID in the community was made, there was still a high number of PWID who are currently receiving health care services (including on MMT and ART) that our study did reach. This will limit the generalizability of the study’s findings. Furthermore, we did not measure how long the participants were given MMT and ART treatment; hence we could not measure the differences between sampling selected from communities or health facilities on HCV and HIV infection status. Additionally, information on duration and frequency of drug injection, which is one of the main injection characteristics, was not captured fully in this study. The previous analysis showed that HCV and HIV infection is positively associated with injection length, and the more extended injection period had higher odds of infection [1, 16]. Moreover, our analysis relies on self-reported lifetime risk behaviors, so it may be subjective due to re-call bias because of a long time period or social desirability. Finally, this study recruited both active and non-active PWID. These two subpopulations might have different risk factors of HCV mono-infection and HIV/HCV co-infection. Further research should consider drug injection characteristics and substance use status when evaluating associated factors of infection status.

## CONCLUSION

Important correlates for HCV mono-infection and HIV/HCV co-infection highlight the need to address risk behaviors of infection and transmission in closed settings such as prisons, and the need to expand the MMT program (to identify those PWID with HCV and HIV), and the importance of establishing HCV sentinel surveillance in order to increase the coverage of HCV testing and treatment. Additionally, this study reported a high prevalence of current HCV and HIV, as well as HCV/HIV co-infection. This, again, shows a demand for HCV treatment and improves access to life-saving hepatitis treatment in Vietnam where affordability makes treatment unavailable to the most PWID.

## Figures and Tables

**Table 1.** Socio-demographic and risk behavior characteristics of surveyed participants.

Characteristics	Total n (%)	Un-infected* n (%)	HCV monoinfection^†^ n (%)	HIV monoinfection^‡^ n (%)	HIV/HCV co-infection^¶^ n (%)
N	509 (100%)	134 (26.33%)	115 (22.59%)	61 (11.98%)	199 (39.10%)
Age, mean (SD)	38.5 (7.17)	37.1 (9.45)	40.9 (7.43)	37.2 (5.52)	38.5 (5.07)
Age Group					
≤35	163 (32.0%)	58 (43.3%)	25 (21.7%)	24 (39.3%)	56 (28.1%)
>35	346 (68.0%)	76 (56.7%)	90 (78.3%)	37 (60.7%)	143 (71.9%)
Gender					
Female	110 (21.6%)	56 (41.8%)	12 (10.4%)	25 (41.0%)	17 (8.5%)
Male	399 (78.4%)	78 (58.2%)	103 (89.6%)	36 (59.0%)	182 (91.5%)
Currently living place					
Sub-urban	95 (18.7%)	18 (13.4%)	24 (20.9%)	6 (9.8%)	47 (23.6%)
Urban	414 (81.3%)	116 (86.6%)	91 (79.1%)	55 (90.2%)	152 (76.4%)
Living Arrangement					
Others	92 (18.1%)	42 (31.3%)	21 (18.3%)	4 (6.6%)	25 (12.6%)
Family	417 (81.9%)	92 (68.7%)	94 (81.7%)	57 (93.4%)	174 (87.4%)
Educational attainment					
Below high school	312 (61.3%)	87 (64.9%)	69 (60.0%)	32 (52.5%)	124 (62.3%)
High school and higher	197 (38.7%)	47 (35.1%)	46 (40.0%)	29 (47.5%)	75 (37.7%)
Marital status					
Separated/Divorced	250 (49.1%)	80 (59.7%)	67 (58.3%)	23 (37.7%)	80 (40.2%)
Married	259 (50.9%)	54 (40.3%)	48 (41.7%)	38 (62.3%)	119 (59.8%)
Currently occupational status					
Unemployed	88 (17.3%)	21 (15.7%)	15 (13.0%)	13 (21.3%)	39 (19.6%)
Employed	421 (82.7%)	113 (84.3%)	100 (87.0%)	48 (78.7%)	160 (80.4%)
Ever Being Incarcerated					
No	194 (38.1%)	64 (47.8%)	28 (24.3%)	35 (57.4%)	67 (33.7%)
Yes	315 (61.9%)	70 (52.2%)	87 (75.7%)	26 (42.6%)	132 (66.3%)
Living with HCV patients					
No	441 (86.6%)	116 (86.6%)	108 (93.9%)	38 (62.3%)	179 (89.9%)
Yes	68 (13.4%)	18 (13.4%)	7 (6.1%)	23 (37.7%)	20 (10.1%)
Ever shared syringes/needles					
No	210 (41.3%)	85 (63.4%)	71 (61.7%)	10 (16.4%)	44 (22.1%)
Yes	299 (58.7%)	49 (36.6%)	44 (38.3%)	51 (83.6%)	155 (77.9%)
Ever reused syringes/needles					
No	330 (64.8%)	99 (73.9%)	91 (79.1%)	34 (55.7%)	106 (53.3%)
Yes	179 (35.2%)	35 (26.1%)	24 (20.9%)	27 (44.3%)	93 (46.7%)
Ever sharing pipe with others					
Yes	240 (47.2%)	78 (58.2%)	54 (47.0%)	19 (31.1%)	89 (44.7%)
No	269 (52.8%)	56 (41.8%)	61 (53.0%)	42 (68.9%)	110 (55.3%)
Having sex with PWID without condoms					
No	326 (64.0%)	80 (59.7%)	87 (75.7%)	22 (36.1%)	137 (68.8%)
Yes	183 (36.0%)	54 (40.3%)	28 (24.3%)	39 (63.9%)	62 (31.2%)
Having sex for money and drug without condoms					
No	396 (77.8%)	110 (82.1%)	94 (81.7%)	50 (82.0%)	142 (71.4%)
Yes	113 (22.2%)	24 (17.9%)	21 (18.3%)	11 (18.0%)	57 (28.6%)
Ever having unsafe tattooing					
No	301 (59.1%)	100 (74.6%)	60 (52.2%)	35 (57.4%)	106 (53.3%)
Yes	208 (40.9%)	34 (25.4%)	55 (47.8%)	26 (42.6%)	93 (46.7%)
Ever sharing personal things (Razor/nail scissors) with others					
No	288 (56.6%)	61 (45.5%)	74 (64.3%)	30 (49.2%)	123 (61.8%)
Yes	221 (43.4%)	73 (54.5%)	41 (35.7%)	31 (50.8%)	76 (38.2%)
HCV knowledge					
Not good (≤5 out of 9)	230 (45.2%)	74 (55.2%)	55 (47.8%)	20 (32.8%)	81 (40.7%)
Good (>5)	279 (54.8%)	60 (44.8%)	60 (52.2%)	41 (67.2%)	118 (59.3%)
Current state of MMT treatment					
No	413 (81.1%)	122 (91.0%)	79 (68.7%)	53 (86.9%)	159 (79.9%)
Yes	96 (18.9%)	12 (9.0%)	36 (31.3%)	8 (13.1%)	40 (20.1%)

* Diagnosed HCV negative and HIV negative;

† Diagnosed HCV positive and HIV negative;

‡ Diagnosed HCV negative and HIV positive;

¶ Diagnosed both HIV and HCV positive.

**Table 2.** Bivariate analysis of factors associated with HCV mono-infection and HCV/HIV co-infection.

	HCV mono-infected^†^ vs. un-infected*		HIV/HCV co-infected^‡^ vs. un-infected		At least one infection^¶^ vs. un-infected	
**Factor**s	OR**	95%CI	OR	95%CI	OR	95%CI
Age Group						
≤35		Ref^††^		Ref		Ref
>35	2.75	1.57–4.81	1.95	1.23–3.09	1.96	1.30–2.96
Gender						
Female		Ref		Ref		Ref
Male	6.16	3.09–12.28	7.69	4.20–14.06	4.26	2.72–6.68
Currently living place						
Sub-urban		Ref		Ref		Ref
Urban	0.59	0.30–1.15	0.50	0.28–0.91	0.60	0.34–1.04
Living Arrangement						
Others		Ref		Ref		Ref
Family	2.04	1.12–3.71	3.18	1.82–5.53	2.97	1.85–4.75
Educational attainment						
Below high school		Ref		Ref		Ref
High school and higher	1.23	0.74–2.07	1.12	0.71–1.77	1.23	0.82–1.86
Marital status						
Single/Separated/Divorced		Ref		Ref		Ref
Married	1.06	0.64–1.76	2.20	1.41–3.44	1.79	1.20–2.67
Currently occupational status						
Unemployed		Ref		Ref		Ref
Employed	1.24	0.61–2.53	0.76	0.43–1.37	0.85	0.50–1.46
Ever Being Incarcerated						
No		Ref		Ref		Ref
Yes	2.84	1.64–4.90	1.80	1.15–2.82	1.72	1.15–2.57
Ever shared syringes/needles						
No		Ref		Ref		Ref
Yes	1.08	0.64–1.80	6.11	3.76–0.30	3.47	2.30–5.23
Ever reused syringes/needles						
No		Ref		Ref		Ref
Yes	0.74	0.41–1.35	2.48	1.54–3.99	1.76	1.14–2.73
Ever sharing a pipe with others						
Yes		Ref		Ref		Ref
No	1.57	0.95–2.60	1.72	1.10–2.68	1.83	1.23–2.73
Having sex with PWID without condoms						
No		Ref		Ref		Ref
Yes	0.48	0.28–0.82	0.67	0.42–1.06	0.78	0.52–1.17
Having sex for money and drug without condoms						
No		Ref		Ref		Ref
Yes	1.02	0.54–1.96	1.84	1.07–3.15	1.43	0.86–2.36
Ever having unsafe tattooing						
No		Ref		Ref		Ref
Yes	2.70	1.58–4.60	2.58	1.60–4.16	2.55	1.64–3.95
Ever sharing personal things (Razor/nail scissors) with others						
No		Ref		Ref		Ref
Yes	0.46	0.28–0.77	0.52	0.33–0.81	0.54	0.37–0.81
HCV knowledge						
Not good (≤5 out of 9)		Ref		Ref		Ref
Good (>5)	1.35	0.82–2.22	1.80	1.15–2.79	1.73	1.16–2.58
The current state of MMT treatment						
No		Ref		Ref		Ref
Yes	4.63	2.27–9.44	2.56	1.29–5.08	2.93	1.55–5.57

* Diagnosed HCV negative and HIV negative;

† Diagnosed HCV positive and HIV negative;

‡ Diagnosed both HCV and HIV positive;

¶ Diagnosed at least either HIV or HCV positive;

** OR: odds ratios and 95% confidence intervals; and

†† ref: reference group.

**Table 3.** Results of the multiple logistic regression analysis of factors associated with HCV mono-infection and HIV/HCV co-infection.

	HCV mono-infected^†^ vs. un-infected*		HIV/HCV co-infected^‡^ vs. un-infected		At least one infection^¶^ vs. un-infected	
	aOR**	95%CI	aOR	95%CI	aOR	95%CI
Being currently on MMT	2.38	1.07 – 5.28	2.11	0.89 – 4.99	2.22	1.08 – 4.56
Ever being incarcerated	2.56	1.33 – 4.90	1.90	1.04 – 3.46	1.59	0.97 – 2.59
Ever shared syringes/needles	1.12	0.55 – 2.27	5.17	2.69 – 9.93	3.42	2.01 – 5.82
Ever reused syringes/needles	0.62	0.27 – 1.41	1.11	0.57 – 2.16	0.96	0.53 – 1.71
Ever sharing a pipe with others	1.75	0.94 – 3.28	2.86	1.55 – 5.29	2.37	1.44 – 3.87
Ever having unsafe tattooing	1.80	0.95 – 3.40	1.61	0.87 – 2.98	1.81	1.09 – 3.03
*Hosmer-Lemesho test (p-value)*	.7547		.4234		.4331	
Area under the curve (AUC)	.8032		.8504		.8040	

* Diagnosed HCV negative and HIV negative;

† Diagnosed HCV positive and HIV negative;

‡ Diagnosed both HCV and HIV positive;

¶ Diagnosed at least either HIV or HCV positive;

** aOR: adjusted odds ratios and 95% confidence intervals;

After adjusting for potential confounders including age group (≤35 vs. >35), gender (male vs. female), marital status (married vs. single/separated/divorced), living arrangement (family vs. others), and HCV knowledge (≤5 vs. >5 out of a maximum 9).

## TAKE-HOME MESSAGE

The current high prevalence of HCV and/or HIV co-infection among Vietnamese PWID shows a heightened need for access to HCV treatment and the importance of establishing HCV sentinel surveillance.

## References

[1] Zhang L, Celentano DD, Minh NL, Latkin C, Mehta S, Frangakis C, Prevalence and correlates of HCV monoinfection and HIV and HCV coinfection among persons who inject drugs in Vietnam. Eur J Gastroenterol Hepatol. 2016;27(5):550–556. Epub 2015/03/15. 10.1097/MEG.0000000000000321 PMC4380662 25769097

[2] Platt L, Easterbrook P, Gower E, McDonald B, Sabin K, McGowan C, Prevalence and burden of HCV co-infection in people living with HIV: a global systematic review and meta-analysis. Lancet Infect Dis. 2016;16(7):797–808. 26922272 10.1016/S1473-3099(15)00485-5

[3] Leung J, Peacock A, Colledge S, Grebely J, Cunningham EB, Hickman M, A global meta-analysis of the prevalence of HIV, hepatitis C virus, and hepatitis B virus among people who inject drugs-Do gender-based differences vary by country-level indicators? J Infect Dis. 2019;220(1):78–90. Epub 2019/02/07. 30726973 10.1093/infdis/jiz058 PMC6775227

[4] Joint United Nations Programme on HIV/AIDS. HIV statistics in Vietnam. 2019 [cited 2019 August 6]. Available from: https://www.unaids.org/en/regionscountries/countries/vietnam.

[5] Berto A, Day J, Van Vinh Chau N, Thwaites GE, My NN, Baker S, Current challenges and possible solutions to improve access to care and treatment for hepatitis C infection in Vietnam: a systematic review. BMC Infect Dis. 2017;17(1):260–270. 28399806 10.1186/s12879-017-2360-6 PMC5387342

[6] Huy BV, Vernavong K, Kính NV. HBV and HCV Coinfection among HIV/AIDS patients in the national hospital of tropical diseases, Vietnam. AIDS Res Treat. 2014;2014:581021. doi:10.1155/2014/581021 25580287 PMC4274838

[7] Tuan NA. Prevalence of HIV/HBV/HCV co-infection among PWID, FSW, MSM in Vietnam. The 6th National scientific conference on HIV/AIDS; Hanoi, Vietnam: Vietnam Authority of AIDS Control; 2017.

[8] Sereno L, Mesquita F, Kato M, Jacka D, Nguyen TTV, Nguyen TN. Epidemiology, responses, and way forward: the silent epidemic of viral hepatitis and HIV coinfection in Vietnam. J Int Assoc Physicians AIDS Care (Chic). 2012;11(5):311–320. 22828983 10.1177/1545109712453939

[9] Baum MK, Jayaweera DT, Duan R, Sales S, Lai S, Rafie C, Quality of life, symptomatology and healthcare utilization in HIV/HCV co-infected drug users in Miami. J Addict Dis. 2008;27(2):37–48. 10.1300/J069v27n02_05 18681190

[10] Fotiou A, Kanavou E, Antaraki A, Richardson C, Terzidou M, Kokkevi A. HCV/HIV coinfection among people who inject drugs and enter opioid substitution treatment in Greece: prevalence and correlates. Hepatol Policy Med. 2016:1–9. 10.1186/s41124-016-0017-5 PMC5918725 30288313

[11] Yen Y-F, Yen M-Y, Su L-W, Li L-H, Chuang P, Jiang X-R, Prevalences and associated risk factors of HCV/HIV co-infection and HCV mono-infection among injecting drug users in a methadone maintenance treatment program in Taipei, Taiwan. BMC Public Health. 2012;12:1066–1073. 23227904 10.1186/1471-2458-12-1066 PMC3534443

[12] Morano J, Gibson B, Altice F. The burgeoning HIV/HCV syndemic in the urban Northeast: HCV, HIV, and HIV/HCV coinfection in an urban setting. PLoS One. 2013;8(5):e64321. 23691197 10.1371/journal.pone.0064321 PMC3653872

[13] Gassowski M, Nielsen S, Bannert N, Bock CT, Bremer V, Ross RS, History of detention and the risk of hepatitis C among people who inject drugs in Germany. Int J Infect Dis. 2019;81:100–106. 30658167 10.1016/j.ijid.2019.01.015

[14] Phan HTT. Hepatitis C and human immunodeficiency virus infections in injecting drug users in drug treatment centers in Vietnam. Scholars Press: University of Texas; 2009.

[15] Nadol P, O’connor S, Duong H, Le L-VN, Thang PH, Tram TH, Findings from integrated behavioral and biologic survey among males who inject drugs (MWID) - Vietnam, 2009–2010: evidence of the need for an integrated response to HIV, hepatitis B virus, and hepatitis C virus. PLoS One. 2015;10(2):e0118304. 25692469 10.1371/journal.pone.0118304 PMC4333571

[16] Do P, Toan T, Van H, Nguyen A, Le G. Prevalence and correlates of hepatitis C virus infection among people who inject drugs in Hanoi 2016. Vietnam J Prev Med. 2018;28(2):45–54.

[17] Martinello M, Hajarizadeh B, Grebely J, Dore GJ, Matthews GV. HCV cure and reinfection among people with HIV/HCV coinfection and people who inject drugs. Curr HIV/AIDS Rep. 2017;14(3):110–121. 28432579 10.1007/s11904-017-0358-8

[18] Rossi C, Butt ZA, Wong S, Buxton JA, Islam N, Yu A, Hepatitis C virus reinfection after successful treatment with direct-acting antiviral therapy in a large population-based cohort. J Hepatol. 2018;69(5):1007–1014. 30142429 10.1016/j.jhep.2018.07.025

[19] Tenner L, Melhado TV, Bobadilla R, Turner BJ, Morgan R. The cost of cure: barriers to access for hepatitis C virus treatment in South Texas. J Oncol Pract. 2019;15(2):61–63. 30668219 10.1200/JOP.18.00525

[20] Akiyama MJ, Cleland CM, Lizcano JA, Cherutich P, Kurth AE. Prevalence, estimated incidence, risk behaviours, and genotypic distribution of hepatitis C virus among people who inject drugs accessing harm-reduction services in Kenya: a retrospective cohort study. Lancet Infect Dis. 2019;19(11):1255–1263. 31540840 10.1016/S1473-3099(19)30264-6 PMC7099605

[21] Yao T, Feng D, Pan MH, Cheng YP, Li CX, Wang J, Related factors and interaction on HIV/HCV co-infection of patients access to methadone maintenance treatment. Zhonghua liuxingbingxue zazhi. 2018;39(5):631–635. 29860807 10.3760/cma.j.issn.0254-6450.2018.05.017

[22] Toro-Tobón D, Berbesi-Fernández D. Prevalence of HIV/Hepatitis C virus co-infection and injection risk correlations in people who inject drugs in Colombia: a cross-sectional study using respondent driven sampling. Subst Use Misuse. 2020;55(3):414–423. 31691646 10.1080/10826084.2019.1683198

[23] Galli M, Ridolfo A, denBogaart Lv, Negri C, Giacomelli A. HCV and drug addiction in the historical scenario of infection entry in Italy. Acta Biomed: Atenei Parmensis. 2018;89(Suppl 10):5–18.

[24] Moradi G, Gouya M-M, Zavareh FA, Bolbanabad AM, Darvishi S, Aghasadeghi MR, Prevalence and risk factors for HBV and HCV in prisoners in Iran: a national bio-behavioural surveillance survey in 2015. Trop Med Int Health. 2018;23(6):641–649. 29698576 10.1111/tmi.13065

[25] Eckhardt B, Winkelstein ER, Shu MA, Carden MR, McKnight C, Jarlais DCD, Risk factors for hepatitis C seropositivity among young people who inject drugs in New York City: Implications for prevention. PLoS One. 2017;12(5):e0177341. 28542351 10.1371/journal.pone.0177341 PMC5438142

[26] Hayashi K, Milloy M-J, Fairbairn N. Incarceration experiences among a community-recruited sample of injection drug users in Bangkok, Thailand. BMC Public Health. 2009;9:492. 20042105 10.1186/1471-2458-9-492 PMC2814810

[27] Vietnam Authority of AIDS Control. Guideline on harm reduction interventions for PWIDs to prevent HIV infection and transmission. Hanoi, Vietnam 2015 [cited 2020 10/13/2019]. Available from: http://vaac.gov.vn/vanban_detail/Detail/Quyet-dinh-01-QD-AIDS-ve-viec-huong-dan-Can-thiep-giam-tachai-du-phong-lay-nhiem-HIV-cho-nguoi-nghien-chich-ma-tuy.

[28] Yi S, Mun P, Chhoun P, Chann N, Tuot S, Mburu G. Prevalence of and risk factors for hepatitis C virus antibody among people who inject drugs in Cambodia: a national biological and behavioral survey. Harm Reduct J. 2019;16(1):29. 31036011 10.1186/s12954-019-0299-1 PMC6489344

[29] Birger RB, Le T, Kouyos RD, Grenfell BT, Hallett TB. The impact of HCV therapy in a high HIV-HCV prevalence population: A modeling study on people who inject drugs in Ho Chi Minh City, Vietnam. PloS one. 2017;12(5):e0177195. 28493917 10.1371/journal.pone.0177195 PMC5426709

[30] Leyna GH, Makyao N, Mwijage A, Ramadhan A, Likindikoki S, Mizinduko M, HIV/HCV co-infection and associated risk factors among injecting drug users in Dar es Salaam, Tanzania: potential for HCV elimination. Harm Reduct J. 2019;16(1):68. 31829199 10.1186/s12954-019-0346-y PMC6907336

